# Supplementary material for: The Systems Biology Research Tool: evolvable open-source software
Source: BMC Syst Biol. 2008 Jun 29;2:55. doi: 10.1186/1752-0509-2-55 (PMC2446383; doi:10.1186/1752-0509-2-55)
Supplement: Additional file 1 — SBRT Archive. An archive of the current version of the Systems Biology Research Tool. [file 1752-0509-2-55-S1.zip › sbrt-1.4.0/doc/developers_guide/api/sbrt/kernel/fba/proc/FluxActivityProcess.Results.html]

FluxActivityProcess.Results


|  |  |  |
| --- | --- | --- |
| **Overview** \| **Package** \| **Class** \| **Use** \| **Tree** \| **Deprecated** \| **Index** \| **Help** | |  |
| **PREV CLASS**   **NEXT CLASS** | **FRAMES**    **NO FRAMES**     **All Classes** |  |
| SUMMARY: NESTED \| FIELD \| CONSTR \| METHOD | DETAIL: FIELD \| CONSTR \| METHOD |  |


---


## sbrt.kernel.fba.proc Class FluxActivityProcess.Results

```
java.lang.Object
  sbrt.kernel.fba.proc.FluxActivityProcess.Results
```

**Enclosing class:**: FluxActivityProcess

---

``` public static class FluxActivityProcess.Results extends java.lang.Object ```

This class is used to represent the results of a flux activity
experiment.

**Author:**
:   This class was written and documented by Jeremiah Wright while in
    the Wagner lab.

---

| **Method Summary** | |
| --- | --- |
| `int` | `active()`             Returns the number of active fluxes. |
| `int` | `inactive()`             Returns the number of inactive fluxes. |

**Methods inherited from class java.lang.Object**

`clone, equals, finalize, getClass, hashCode, notify, notifyAll, toString, wait, wait, wait`

**Method Detail**

### active

```
public int active()
```

:   Returns the number of active fluxes.

    :   **Returns:**: the number of active fluxes.

---


### inactive

```
public int inactive()
```

:   Returns the number of inactive fluxes.

    :   **Returns:**: the number of inactive fluxes.


---


|  |  |  |
| --- | --- | --- |
| **Overview** \| **Package** \| **Class** \| **Use** \| **Tree** \| **Deprecated** \| **Index** \| **Help** | |  |
| **PREV CLASS**   **NEXT CLASS** | **FRAMES**    **NO FRAMES**     **All Classes** |  |
| SUMMARY: NESTED \| FIELD \| CONSTR \| METHOD | DETAIL: FIELD \| CONSTR \| METHOD |  |


---
